# Emergence of Rhino-Orbito-Cerebral Mucormycosis in Peru: Impact of the COVID-19 Pandemic

**DOI:** 10.7759/cureus.45240

**Published:** 2023-09-14

**Authors:** Robert Cumpa-Quiroz, Federico Elguera-Falcón, David R Guevara-Lazo

**Affiliations:** 1 Department of Internal Medicine, Hospital Nacional Arzobispo Loayza, Lima, PER; 2 School of Medicine, Universidad Peruana Cayetano Heredia, Lima, PER

**Keywords:** type 2 diabetes mellitus, zygomycosis, sars-cov-2, covid-19, mucormycosis

## Abstract

Introduction

Mucormycosis is an invasive fungal infection caused by Mucorales that has been increasingly recognized over the years, particularly during the COVID-19 pandemic. Nevertheless, in Peru, there has been limited research on this disease. This study seeks to provide insights into the demographics, clinical presentations, treatment, and outcomes of patients with mucormycosis, before and during the COVID-19 pandemic.

Materials and methods

We conducted a retrospective case series by reviewing the medical records of Peruvian patients hospitalized at a referral medical center between 2017 and 2021. The selection criteria included patients aged 18 years or older with clinical features of rhino-orbito-cerebral mucormycosis supported by radiological imaging. We extracted data related to risk factors for mucormycosis infection, clinical presentation, management, and hospitalization. Data analysis was performed using Stata software (StataCorp LLC, College Station, Texas, USA) to compare patient groups before and during the COVID-19 pandemic.

Results

Nineteen cases met our selection criteria: 11 men and eight women with an average age of 57.6 ± 10.6 years. All 19 patients had type 2 diabetes mellitus as comorbidity, with 13 cases exhibiting uncontrolled diabetes. Six patients presented before the COVID-19 pandemic, while 13 during its course. Within the group of patients diagnosed during the pandemic, nine were diagnosed with SARS-CoV-2 infection. Regarding the site of mucormycosis infection, the paranasal sinuses were predominantly involved. Survival analysis indicated that patients who developed mucormycosis during the COVID-19 pandemic, those with uncontrolled diabetes, or those who did not undergo surgery had lower probabilities of survival.

Conclusion

Mucormycosis is a rare infection associated with high mortality and morbidity with increased frequency during the COVID-19 pandemic. Early diagnosis, timely administration of antifungal treatment, surgery, and effective management of comorbidities can have life-saving implications. Unfortunately, despite the availability of various diagnostic tests and less toxic antifungal options such as liposomal amphotericin-B, such resources are not accessible in Peru's national hospitals.

## Introduction

Mucormycosis is an angio-invasive fungal infection caused by organisms of the Mucorales order, which include the genera Rhizopus, Mucor, Rhizomucor, etc. [1]. These fungi are saprophytes and have a worldwide distribution, with Rhizopus spp. being the predominant genus in South America and more associated with rhino-orbito-cerebral mucormycosis (ROCM) [2]. The appearance of the COVID-19 pandemic has caused an increase in the burden of ROCM around the world. This upward trend was higher in India than in other countries [3,4]. In Peru, ROCM has been poorly studied with research papers primarily consisting of case reports [5]. Therefore, our objective is to provide valuable information regarding the demographic, clinical profile, treatment, and outcome of patients with ROCM before and during the COVID-19 pandemic from a tertiary medical center in Peru.

## Materials and methods

Study population and clinical assessment

A retrospective case series review of medical records was undertaken at the Hospital Nacional Arzobispo Loayza, with the aim of identifying patients diagnosed with ROCM between 2017 and 2021. The patients were initially identified under the hospital’s registration code B46.1 in accordance with the International Classification of Diseases 10th Edition. The selection criteria encompassed patients aged 18 years or older, presenting signs and symptoms consistent with ROCM supported by radiological imaging (i.e. computed tomography or magnetic resonance) or nasal endoscopy. Patients with incomplete medical records were excluded. Incomplete medical records were defined as the absence of information regarding diagnosis, clinical presentation, comorbidities, or management. Data of the most frequent risk factors were registered: diabetes mellitus type 2 (DM2), SARS-CoV-2 infection, and the use of glucocorticoids. Additionally, information about time to hospital admission, length of hospital stay, surgical management, antifungal therapy, adverse effects of antifungal therapy, and outcomes was extracted. Diabetes mellitus was categorized as "controlled" if the glycosylated hemoglobin level was <8%, and as "uncontrolled" if it was ≥8% [6]. SARS-CoV-2 infection was detected through a positive antigen test. Moreover, patients were classified into symptomatic and asymptomatic groups based on their clinical presentation of COVID-19.

Data analysis

Data entry was conducted using Excel, and subsequent statistical analyses and graphics were carried out using Stata software (Release 17; StataCorp LLC, College Station, Texas, USA), with the software license provided by the Universidad Peruana Cayetano Heredia. To provide a comprehensive overview of the data, descriptive statistics were employed. The results were presented in the form of either medians and interquartile ranges or means and standard deviations, depending on the normality distribution, which was assessed using the Shapiro-Wilk test. Categorical variables were summarized using counts and percentages. For a comparative analysis, bar graphs and box plots were generated to illustrate the frequency and age, respectively, in both groups before and during the COVID-19 pandemic. Survival analysis was conducted with death serving as the censoring variable and Kaplan-Meier curves were plotted to depict the cumulative probability of survival at different time points.

## Results

Demographic data

Nineteen medical records that met the selection criteria were identified (supplemental table). The final group consisted of eleven men and eight women, with an average age of 57.6 ± 10.6 years, as shown in Table 1. Twelve patients were residing in Lima, the capital city of Peru, while seven patients had been referred to our hospital from other cities. In 15 cases, the diagnosis was confirmed through microbiological examinations involving histopathological analysis, while in four cases, the diagnosis was clinical and radiographic. In our series, there were six cases before the COVID-19 pandemic and 13 during its course.

**Table 1:** Characteristics and Outcomes of Rhino-Orbito-Cerebral Mucormycosis Patients †Mean ± st. dev. *Median (min-max)

	Before the COVID-19 pandemic (n=6)	%	During the COVID-19 pandemic (n=13)	%	Overall (n=19)	%
Age	66.5 ± 9.0†		53.5 ± 8.9†		57.6 ± 10.6†	
41-59 years	2	33.3	10	76.9	12	63.2
60-81 years	4	66.7	3	23.1	7	36.8
Sex						
Female	4	66.7	4	30.8	8	42.1
Male	2	33.3	9	69.2	11	57.9
City of origin						
Lima	2	33.3	10	76.9	12	63.2
Other cities	4	66.7	3	23.1	7	36.8
Diabetes mellitus type 2						
Controlled	2	33.3	4	30.8	6	31.6
Uncontrolled	4	66.7	9	69.2	13	68.4
COVID-19						
Negative	6	100.0	4	30.8	4	21.1
Asymptomatic	0	0.0	3	23.1	3	15.8
Symptomatic	0	0.0	6	46.2	6	31.6
Received corticosteroids						
Yes	1	16.7	0	0.0	1	5.3
No	5	83.3	13	100.0	18	94.7
ROCM stage						
Stage 2	2	33.3	2	15.4	4	21.1
Stage 3	3	50.0	6	46.2	9	47.4
Stage 4	1	16.7	5	38.5	6	31.6
Time to hospital admission	14.5 (5-120)*		14 (3-60)*		14 (3-120)*	
Length of hospital stay	50.6 ± 25.1†		40.5 ± 34.5†		43.7 ± 31.5†	
Outcome						
Medical discharge	6	100.0	9	69.2	15	78.9
Deceased	0	0.0	4	30.8	4	21.1

Impact of the COVID-19 pandemic

**Figure 1:**
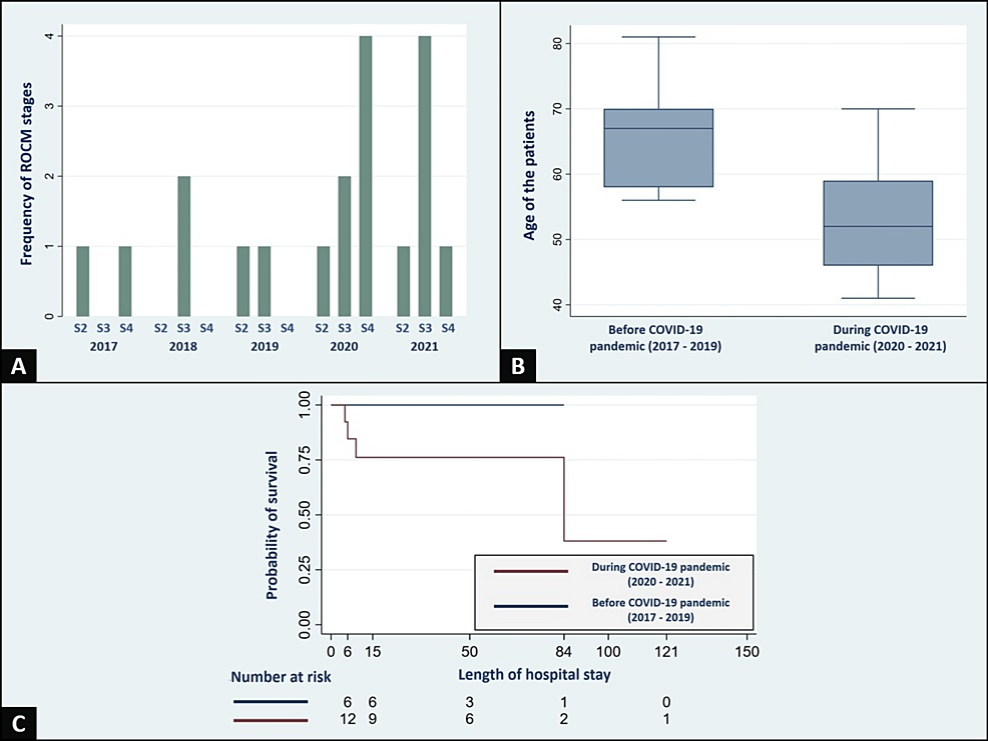
A Comparative Analysis of Mucormycosis Before and During the COVID-19 Pandemic In Panel A, the rising frequency of rhino-orbito-cerebral mucormycosis cases is evident from 2017 to 2021, with a notable frequency of severe cases (stages 3 and 4) during the COVID-19 pandemic. Panel B presents a box plot illustrating the contrast in ages among rhino-orbito-cerebral mucormycosis cases before and during the COVID-19 pandemic. Panel C displays the Kaplan-Meier survival curve, comparing the estimated survival rates of patients with mucormycosis before and during the pandemic.

During the COVID-19 pandemic, an increase in the annual frequency of recorded ROCM cases occurred, with a predominance of severe stages 3 and 4, as visualized in Figure 1A. Furthermore, patients displayed a notable gender and age trend shift. They were predominantly male (69.2% vs. 33.3%) and comparatively younger with a mean age of 53.5 ± 8.9 years, contrasting the pre-COVID-19 era (mean age of 66.5 ± 9.0 years), as depicted in Figure 1B. Figure 1C illustrates the COVID-19 pandemic's impact on mortality through Kaplan-Meier survival curves. The probability of survival during this period was diminished, measuring at 85% (95% CI: 64%-97%) on day six, 76% (95% CI: 58%-94%) on day 15, and 38% (95% CI: 9%-86%) on day 84.

Clinical data

**Figure 2:**
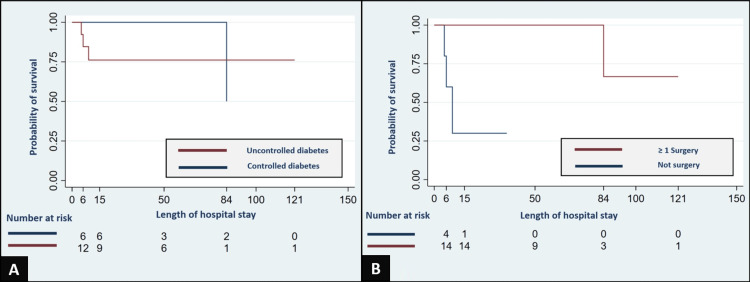
Impact of Diabetes Control and Surgical Intervention In Panel A, a Kaplan-Meier survival curve illustrates a comparison of estimated survival rates for patients with mucormycosis based on the presence of uncontrolled and controlled type 2 diabetes mellitus at the time of admission. Panel B showcases a Kaplan-Meier survival curve comparing estimated survival rates for patients with mucormycosis who received at least one surgery with those who did not undergo surgery.

All cases had DM2 as a comorbidity, and among them, 13 cases exhibited uncontrolled DM2. We found that patients with uncontrolled DM2 faced a diminished probability of survival, measuring 84% (95% CI: 51%-95%) on day six and 76% (95% CI: 43%-91%) on day 15 (Figure 2A). In the group of 16 patients with ROCM during the COVID-19 pandemic, nine of them were diagnosed with SARS-CoV-2 infection. Of these, six cases were symptomatic, while three remained asymptomatic. Notably, one case experienced severe SARS-CoV-2 infection and subsequently underwent corticosteroid treatment as part of the management (Table 2). Regarding mucormycosis infection, the nasal mucosa and paranasal sinuses were the predominant areas of mucormycosis infection (95%), followed by the orbit (79%), the central nervous system (32%), and the oral cavity (26%).

**Table 2:** Antifungal Treatment and Surgical Management for Rhino-Orbito-Cerebral Mucormycosis Patients †Mean ± st. dev. *Median (min-max)

	Before the COVID-19 pandemic (n=6)	%	During the COVID-19 pandemic (n=13)	%	Overall (n=19)	%
Surgical management	1.6 ± 1.2†		1.8 ± 1.5†		1.8 ± 1.4†	
No	1	16.7	4	30.8	5	26.3
Yes	5	83.3	9	69.2	14	73.7
Antifungal therapy						
Received isavuconazole and amphotericin	0	0.0	4	30.8	4	21.1
Received isavuconazole	0	0.0	1	7.7	1	5.3
Received amphotericin	6	100.0	8	61.5	14	73.7
Amphotericin cumulative dose	1,462.5 (172-3,700)*		450 (0-5150)*		1,200 (0-5,150)*	
Amphotericin side effects						
No	2	33.3	3	23.1	5	26.3
Yes	4	66.7	10	76.9	14	73.7

The analysis of mucormycosis-related hospitalizations indicated that the median time for hospital admission was 14 days (IQR, 5-45 days; range 3-120 days). In contrast, the mean hospital stay averaged 43.7 ± 31.5 days (range 5-121 days). Additionally, a readmission rate of 15% was observed.

Antifungal and surgical management

The management is summarized in Table 2. Deoxycholate amphotericin-B was the preferred antifungal in 18 cases, with administration through a central venous catheter in 75% of cases and via the peripheral route in 25%. One case was exclusively treated with isavuconazole, whereas four patients received a combination of amphotericin-B and isavuconazole. The median cumulative dose of deoxycholate amphotericin-B administered was 1225 mg (IQR 250-1,650 mg; ranging from 0 to 5,150 mg). The adverse effects associated with antifungal treatment included anemia (68%), hypokalemia (42%), and azotemia (32%). All of these complications were resolved with appropriate management. Surgical management was performed on 14 patients, while five of them did not undergo surgery. The survival analysis revealed that patients who did not undergo surgery faced a lower probability of survival, measuring at 60% (95% CI: 13%-88%) on day six and 30% (95% CI: 1%-71%) on day 15 (Figure 2B).

## Discussion

Epidemiology

The prevalence of ROCM varies across countries. In the United States, hospitalization prevalence was reported as 0.16 per 10,000 discharges, while in India, it is more frequent, with a prevalence of 0.14 per 1,000 inhabitants [7,8]. Most cases of ROCM have at least one risk factor. In our study, uncontrolled DM2, SARS-CoV-2 infection, and the use of glucocorticoids were the most common, as shown in Table 1. A retrospective study conducted in México, a country with a population similar to Peru's, reported uncontrolled DM2 as the most frequent risk factor among ROCM patients. However, other documented risk factors include long-term glucocorticoid therapy, DM2 complicated with ketoacidosis, hematologic malignancies, neutropenia, hematopoietic cell transplantation, iron overload, deferoxamine therapy, chronic kidney disease, and a past medical history of tuberculosis [1,2,4,9,10].

The incidence of ROCM has increased globally during the COVID-19 pandemic [3]. Our study also highlights a notable surge in cases during this period. One possible explanation is that SARS-CoV-2 infection creates an ideal environment of hypoxemia, hyperglycemia, acidity, increased ferritins, reduced white blood cell count, and phagocytic activity (due to SARS-CoV-2-related immunosuppression, use of corticosteroids, or pre-existing conditions) [8], along with the widespread use of antibiotics, which favors the development of the infection.

Clinical presentation

**Figure 3:**
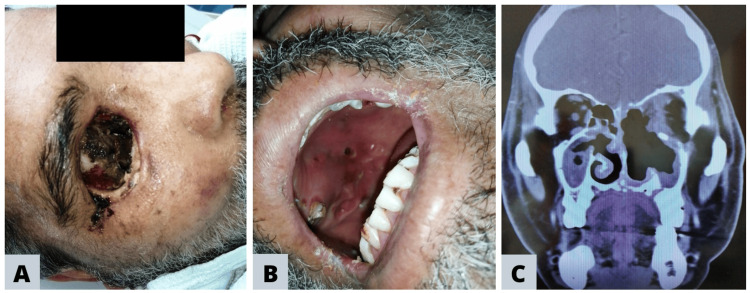
Clinical and Radiological Manifestations of Rhino-Orbital-Cerebral Mucormycosis Panels A and B depict the case of a 51-year-old male patient with stage 3 rhino-orbital-cerebral mucormycosis and uncontrolled diabetes (case number 16, supplemental material). In Panel A, the orbital basin displays inflammatory and necrotic tissue prior to surgical cleaning. Panel B highlights multiple necrotic ulcer lesions on the palate and teeth in a state of poor preservation and hygiene. Panel C offers a coronal view of computed tomography from a 58-year-old male patient with stage 4 rhino-orbital-cerebral mucormycosis and uncontrolled diabetes (case number 8, supplemental material). The image reveals the left maxillary sinus with dense heterogeneous tissue, lysis of the medial and posterior walls, frontal erosive bone lesions, and erosion of the lamina propria.

Mucorales infections exhibit a wide spectrum of clinical presentations, encompassing pulmonary, cutaneous, gastrointestinal, and disseminated mucormycosis. Nonetheless, ROCM stands as the predominant form (Figure 3), primarily affecting the nasal mucosa and, to a lesser extent, involving the central nervous system (CNS). At present, a standardized classification for evaluating ROCM remains absent. However, Dr. Honavar introduced a staging system that follows the anatomical progression of the infection from nasal mucosa to CNS [11]. The demographic trend we observed is that ROCM emerged among younger patients during the COVID-19 pandemic (Figure 1B), along with a surge in the frequency of complicated ROCM cases, stages 3 and 4, as depicted in Figure 1A. This occurrence is possibly influenced by the aggressive nature of mucormycosis infection, compounded by the interplay of risk factors such as SARS-CoV-2 infection, uncontrolled DM2, and limited access to specialized medical care during the COVID-19 pandemic.

Diagnosis

ROCM represents an invasive fungal infection with significant mortality and morbidity, underscoring the urgency of early diagnosis to enhance patient prognosis. Furthermore, it is important to include ROCM in the differential diagnosis of complex sinusitis among diabetic patients [12]. The diagnosis of ROCM entails clinical suspicion, imaging, and histopathological analysis. Techniques such as nasal endoscopy, magnetic resonance imaging, or computed tomography are employed to assess paranasal sinus, orbit, and CNS involvement, as well as to evaluate the extension. In our series, histopathological analysis confirmed 15 cases, while four cases were diagnosed clinically and radiologically due to challenges associated with isolating and identifying the fungus, stemming from tissue sample contamination and necrosis.

Management

The management of ROCM requires a multidisciplinary approach, fostering collaboration between the treating physician and the surgical team. Regarding antifungal therapy, the prompt initiation of antifungals assumes paramount importance, as it influences the patient's prognosis. A retrospective study has underscored the impact of delayed amphotericin-B therapy beyond six days after diagnosis, which resulted in a twofold increase in mortality rates [13].

Liposomal amphotericin-B stands as the preferred antifungal choice for ROCM due to its reduced toxicity [14]. However, within our setting, the lipid formulation remains unavailable; hence, the patients received amphotericin-B deoxycholate. Additionally, five cases were treated with isavuconazole due to the unavailability of amphotericin-B deoxycholate. The administration of amphotericin-B deoxycholate can be conducted through a central venous catheter or a peripheral venous catheter. In our series, peripheral administration yielded no severe adverse effects. This approach holds significance in the context of potential delays in central venous catheter placement, which can impede the timely initiation of treatment.

The cases who did not undergo surgical intervention faced a decreased probability of survival (Figure 2B). This aligns with findings from a retrospective study that showcased a correlation between the use of antifungal therapy in conjunction with surgical debridement and lower mortality rates, as opposed to individuals who solely received one of the interventions [15].

Mucormycosis-related hospitalizations

Beyond its high mortality and morbidity, ROCM contributes to prolonged hospitalizations and delayed time to admission. Our study revealed a median time to hospital admission of 14 days, with a considerable range spanning from three to 120 days. This observation underscores the restricted accessibility to specialized medical care for individuals of lower socioeconomic status in Peru. Moreover, our findings indicate a mean hospital stay duration of 43.7 days, a result potentially stemming from multifaceted factors. These factors could encompass the infection's aggressive nature, the management of underlying comorbidities, the elevated risk of possible complications, and the time required for recovery and rehabilitation.

Limitations

Our study presents several limitations that warrant consideration while interpreting the results. Many of these stem from the retrospective design and the small sample size, thereby posing challenges in generalizing the findings. Moreover, we did not gather data on variables such as COVID-19 vaccination, use of antibiotics, diabetes-related complications like ketoacidosis, and chronic kidney disease, all of which could potentially impact prognosis. Nonetheless, it is crucial to acknowledge that mucormycosis remains an uncommon infection, and this study stands as the most comprehensive series on mucormycosis among Peruvian patients.

## Conclusions

Mucormycosis is a rare and challenging disease to diagnose, characterized by high morbidity and mortality rates. Its incidence has shown an upward trend, particularly peaking during the COVID-19 pandemic. Recognizing this potentially fatal condition is crucial for healthcare providers, as prompt surgical intervention, antifungal treatment, and effective management of underlying comorbidities can be life-saving. Despite the existence of readily accessible diagnostic tests and less toxic therapeutic options such as liposomal amphotericin-B, these resources are often unavailable in Peru's national hospitals. While our understanding of mucormycosis has improved following the COVID-19 pandemic, survival rates for this condition still remain disappointingly low.

## Appendices

**Table 3:** Detailed Information of Rhino-Orbital-Cerebral Mucormycosis Patients: Demographics, Clinical Profiles, Management, and Outcomes We employed the case definitions and staging proposed by Honavar for ROCM [11]. Optimal management included initiating antifungal treatment and surgical intervention within the first 24 hours of the patient's admission. In Case 5, a total of 1,000 mg of intravenous amphotericin-B was administered, supplemented with 25 mg/day applied directly into the nostrils for 11 days, resulting in a cumulative dose of 1,275 mg. In Case 9, dexamethasone was administered at a daily dose of 6 mg for 10 days as part of the treatment for severe COVID-19. In Case 10, intravenous isavuconazole treatment was administered for 30 days at a daily dose of 200 mg, and amphotericin-B was not administered. DM2, Diabetes mellitus type 2; ROCM, rhino-orbital-cerebral mucormycosis

Case	Date of admission	Case definition	State-wise distribution of ROCM cases	Sex	Age	Type 2 diabetes mellitus	SARS-CoV-2 infection	Staging of ROCM	Time to hospital admission	Length of hospital stay	Initiation of antifungal treatment	Route of administration of amphotericin-B	Accumulated dose of amphotericin-B (mg)	Treatment with isavuconazole	Surgical management	Adverse effects	Outcome
Before COVID-19 pandemic																	
1	1/8/2017	Proven	Ancash	M	81	Uncontrolled DM2	Not apply	Stage 4: Involvement of the orbit and the CNS	15	73	Optimal	Peripheral line	1650	No	Optimal and 3 surgeries were needed	Not reported	Medical discharge
2	7/3/2018	Proven	Ucayali	M	66	Uncontrolled DM2	Not apply	Stage 3: Involvement of the nose, paranasal sinuses, and the orbit	20	53	Late, at 7 days	Central line	2250	No	Late, on day 8, and 2 surgeries were needed	Azotemia, hypokalemia, and anemia	Medical discharge
3	8/4/2018	Proven	Ica	F	70	Controlled DM2	Not apply	Stage 2: Involvement of the nose and the paranasal sinuses	120	15	Optimal	Central line	1250	No	Late and 1 surgery was needed	Anemia	Medical discharge
4	25/4/2018	Proven	Lima	F	68	Uncontrolled DM2	Not apply	Stage 3: Involvement of the nose, paranasal sinuses, and the orbit	5	43	Late, at 6 days	Central line	172	No	Optimal and 3 surgeries were needed	Not reported	Readmission
5	24/3/2019	Proven	Lima	F	58	Uncontrolled DM2	Not apply	Stage 3: Involvement of the paranasal sinuses, orbit, and oral cavity	7	36	Late, at 6 days	Central line	1275	No	None	Azotemia and anemia	Medical discharge
6	7/12/2019	Proven	Ancash	F	56	Controlled DM2	Not apply	Stage 2: Involvement of the paranasal sinuses and oral cavity	14	84	Optimal	Central line	3700	No	Late and 1 surgery was needed	Azotemia, hypokalemia, and anemia	Medical discharge
During COVID-19 pandemic																	
7	7/8/2020	Probable	Lima	F	44	Uncontrolled DM2	Symptomatic	Stage 4: Involvement of the nose, orbit, and the CNS	10	5	Optimal	Central line	250	No	None	Hypokalemia	Deceased
8	7/8/2020	Proven	Lima	M	58	Uncontrolled DM2	Asymptomatic	Stage 4: Involvement of the nose, oral cavity, orbit, and the CNS	45	59	Optimal	Central line	1500	Treated during 21 days	Late, on day 15, and 2 surgeries were needed	Azotemia, hypokalemia, and anemia	Medical discharge
9	10/9/2020	Proven	Lima	M	45	Uncontrolled DM2	Symptomatic	Stage 4: Involvement of the nose, oral cavity, orbit, and the CNS	44	8	Optimal	Central line	250	Treated during 3 days	None	Anemia	Readmission
10	18/9/2020	Proven	Cajamarca	M	46	Controlled DM2	Negative	Stage 4: Involvement of the nose, orbit, and the CNS	14	30	Optimal	Did not receive amphotericin B	Did not receive amphotericin B	Treated during 30 daysᵉ	Optimal and 4 surgeries were needed	Not reported	Medical discharge
11	1/12/2020	Proven	Lima	M	67	Uncontrolled DM2	Negative	Stage 3: Involvement of the nose, paranasal sinuses, and the orbit	4	121	Optimal	Central line	5150	Treated during 35 days	Late, on day 8, and 3 surgeries were needed	Hypokalemia and anemia	Medical discharge
12	14/12/2020	Proven	Callao	F	70	Uncontrolled DM2	Asymptomatic	Stage 3: Involvement of the nose and the orbit	5	9	Optimal	Peripheral line	350	No	None	Not reported	Deceased
13	25/1/2021	Probable	Lima	M	53	Controlled DM2	Symptomatic	Stage 2: Involvement of the paranasal sinuses	60	18	Optimal	Central line	850	No	Late and 3 surgeries were needed	Not reported	Medical discharge
14	7/4/2021	Proven	Lima	M	52	Uncontrolled DM2	Symptomatic	Stage 2: Involvement of the paranasal sinuses and oral cavity	60	54	Optimal	Central line	1200	No	Late, on day 27, and 1 surgery was needed	Hypokalemia and anemia	Medical discharge
15	16/4/2021	Proven	Lima	F	50	Controlled DM2	Symptomatic	Stage 3: Involvement of the paranasal sinuses and the orbit	7	50	Late, at 2 days	Central line	450	No	Late and 3 surgeries were needed	Anemia	Medical discharge
16	4/5/2021	Probable	La Libertad	M	51	Uncontrolled DM2	Negative	Stage 3: Involvement of the nose and the orbit	5	50	Optimal	Peripheral line	2350	No	Late and 3 surgeries were needed	Azotemia, hypokalemia, and anemia	Readmission
17	20/5/2021	Proven	Lima	M	41	Uncontrolled DM2	Negative	Stage 3: Involvement of the nose and the orbit	3	33	Optimal	Peripheral line	160	No	Late and 2 surgeries were needed	Anemia	Medical discharge
18	20/5/2021	Proven	Lima	M	59	Controlled DM2	Symptomatic	Stage 4: Involvement of the nose, orbit, and the CNS	59	84	Optimal	Central line	1550	Treated during 5 days	Late, on day 27, and 2 surgeries were needed	Azotemia, hypokalemia, hypomagnesemia, and anemia	Deceased
19	16/6/2021	Probable	Lima	F	60	Uncontrolled DM2	Asymptomatic	Stage 3: Involvement of the nose and the orbit	21	6	Late, at 4 days	Central line	100	No	None	Anemia	Deceased
